# Oxic Fe(III) reduction could have generated Fe(II) in the photic zone of Precambrian seawater

**DOI:** 10.1038/s41598-018-22694-y

**Published:** 2018-03-09

**Authors:** Elizabeth D. Swanner, Markus Maisch, Wenfang Wu, Andreas Kappler

**Affiliations:** 1 Department of Geological and Atmospheric Sciences, Iowa State University, Ames, IA United States of America; 2 Department of Geosciences, University of Tuebingen, Tuebingen, Germany; 3 Key Laboratory of Earth and Planetary Physics, Institute of Geology and Geophysics, Chinese Academy of Sciences, Beijing, China

**Keywords:** Water microbiology, Element cycles, Geochemistry, Marine chemistry, Marine chemistry

## Abstract

Many marine Precambrian iron formations (IF) record deep anoxic seawater enriched in Fe(II) (i.e. ferruginous) overlain by mildly oxygenated surface water. This is reflected by iron-rich sediments forming in deep basins, and relatively iron-poor sediments forming in shallow, sunlit waters. Such an iron gradient is often interpreted as a redox interface where dissolved Fe(II) was oxidized and precipitated as Fe(III)-bearing minerals. As such, sedimentary iron enrichments are proxy to the progressive oxidation of the oceans through geological time. However, this interpretation is founded on the assumption that Fe(II) could not persist within an oxygenated water column. Here, we cultivated cyanobacteria in an illuminated column supplied with Fe(II)-rich seawater medium in a laboratory-scale analog of a continental margin supporting IF deposition. We first observed Fe(II) oxidation with oxygen, then biologically-mediated reduction of Fe(III) (oxyhydr)oxides, which maintained a pool of Fe(II) in the presence of oxygen. Such steady-state iron redox cycling may have maintained dissolved, and hence mobile Fe(II) in oxygenated seawater above ferruginous deep basins such as those inferred for many Precambrian IF.

## Introduction

Interfaces between deep, anoxic and Fe(II)-containing (i.e. ferruginous) seawater and overlying oxygenated surface water existed for billions of years on Earth^1–4^. Due to the low redox potential of Fe^2+^/Fe^3+^ (E°′ = +0.36 V), non-diagenetic sedimentary enrichments indicative of Fe(II) in seawater should indicate the absence of oxygen in that same water. This delineation between oxic and ferruginous water masses manifests itself as a sharp redoxcline between Fe(II) and oxygen-containing layers of water in modern anoxic basins and lakes, or in sediment cores, such that Fe(II) and oxygen are generally not observed to coexist in stratified systems^5,6^.

After Fe(II) is oxidized and precipitated, generally as Fe(III) (oxyhydr)oxide minerals under circumneutral pH conditions, it can be reduced back to Fe(II) by the activity of dissimilatory Fe(III)-reducing bacteria within anoxic sediments containing organic carbon, regenerating Fe(II). Alternatively, Fe(III) minerals can be reductively dissolved by hydrogen sulfide^7^, formed after microbial sulfate reduction in anoxic sediments supplied with sufficient organic carbon. However, if oxygenated waters are present over anoxic and/or sulfidic sediments, remobilized Fe(II) should not re-enter the water column, as it will be rapidly re-oxidized at the oxic-anoxic interface^5^, effectively immobilizing iron in oxygenated settings. There are, however, photochemical and enzymatic pathways for Fe(III) reduction that occur in the presence of oxygen, and are known to generate steady-state pools of Fe(II) within the modern ocean^8,9^.

One such pathway is photochemical Fe(III) reduction, which abiotically transfers electrons from Fe(III)-binding organic ligands bound to Fe(III), forming Fe(II) in a process termed ligand-to-metal charge transfer (LMCT)^10^. Complexation to organic ligands changes the stability of Fe(III) and thus its accessibility to redox transformations^11^. Light also induces the production of reactive oxygen species (ROS) from organics or from the splitting of water. ROS react with iron, and can result in Fe(III) reduction and Fe(II) oxidation^11,12^. Cyanobacteria, the lineage of modern bacteria thought to be related to Earth’s earliest photosynthetic oxygen-producers, have been shown to enzymatically reduce Fe(III) directly^13^, as well as via enzymatic production of ROS^14^, which then reduce Fe(III), with both processes occurring under oxic conditions. Thus, within sunlit water, shallow microbial mats, or sediments there is the possibility for oxic Fe(III) reduction by processes that are distinct from dissimilatory microbial Fe(III) reduction or reductive dissolution occurring in anoxic sediments. Furthermore, these processes for Fe(III) reduction may have been favored by the higher concentrations of organic material available to complex and solubilize Fe(III) in the upper water column of Precambrian seawater^15^.

It is unclear from studies of oxic Fe(III) reduction in modern systems, however, whether such processes could occur under the range of Fe(II) and oxygen concentrations relevant for Precambrian IF depositional settings. Furthermore, can oxic Fe(III) reduction generate Fe(II) in high enough abundance to be recorded in the sedimentary iron record? This would require steady-state reduction of Fe(III) to Fe(II), such that the Fe(II) is continually produced and persists despite rapid re-oxidation with oxygen. Finally, can oxic Fe(III) reduction permit transport of dissolved Fe(II) within suboxic or oxic environments, such that sediments might record the presence of Fe(II) in oxygenated seawater?

**Figure 1:**
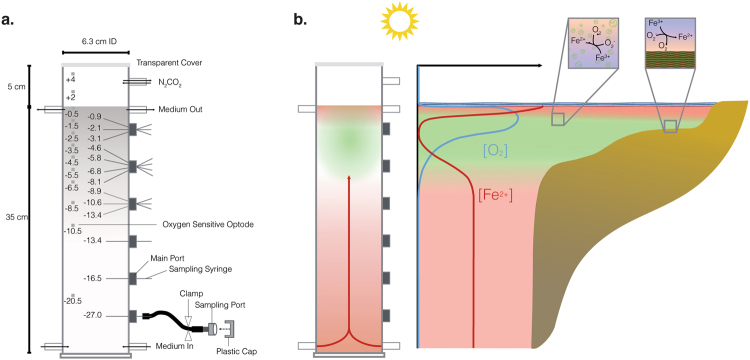
(**a**) Schematic cross-section and dimensions of the column. Small darker grey squares are optodes, labeled with the depth in the column. Dark grey sampling ports show the arrangement of fixed needles used to take aqueous samples at different depths in the center of the column. White ports show where medium was pumped in (bottom), pumped out (top), and an additional port used for flushing the headspace. (**b**) Schematic cross-section of a Precambrian continental margin overlying a deep, ferruginous basin, simulated in one dimension (vertically) by the column experiment. Insets show that oxic Fe(III) reduction described here could take place in the water column and/or in a benthic setting, simulated by glass beads in the column.

To address these questions, we utilized a flow-through column where a marine planktonic cyanobacterium was grown in a gradient of Fe(II)-rich seawater medium advectively supplied from the bottom, and light provided from the top^16^. This column is a laboratory-scale analogue to both the water column and shallow microbial mat environments that would have been present along a Precambrian continental margin exposed to upwelling Fe(II)-rich waters (Fig. 1). Over 4 weeks of column deployment, we tracked depth-dependent Fe(II), Fe(III), and oxygen concentrations in the presence of a marine cyanobacterium, *Synechococcus* PCC 7002. The results are used to address the possibility of a dynamic iron redox cycle within an oxygenated surface ocean above an anoxic and ferruginous deep basin, such as those from which Precambrian IF were deposited.

## Methods

Extensive details of column design, setup, and operation have been published^16^, and so are described only briefly here. The column was a modified 1 L glass graduated cylinder (Fig. 1). Two inflow ports were added at the bottom of the column, and two at the top, resulting in about 35 cm of water depth. Six sampling ports were distributed every 5 cm vertically throughout the column, and closed with butyl rubber stoppers. The column was filled with 0.5 to 0.7 mm glass beads (porosity 0.379), used to help stabilize chemical gradients against disturbance from any external vibrations. Anoxic marine phototroph (MP) medium, which is a seawater medium that utilizes a 22 mM sodium bicarbonate buffer^17^, was prepared to have an initial Fe(II) concentration of ca. 400 µM Fe(II)Cl_2_ and a pH of 6.8, although actual Fe(II) concentration varied following precipitation of Fe(II) carbonate and phosphate mineral phases, which were removed by filtration prior to use. It was pumped into the bottom two inflow ports through rubber tubing connected to butyl rubber-stoppered media bottles, and exited the top ports into empty media bottles (Fig. 1). The 5 L medium reservoir was replaced on days 8 and 17. The Fe(II) concentration was 389 μM in the first medium bottle (days 0–8), 307 μM (days 8–17), and 302 μM (days 17 to 30). N_2_/CO_2_ (v/v, 90/10, 10 mbar) was used to initially flush the pore space of the column, and the headspace of the column was flushed continuously to simulate an anoxic atmosphere.

A light source (Samsung SI-P8V151DB1US) was installed 2 cm above the column, which created a light gradient within the upper 4 cm (Supplementary Information). The spectrum of the light source, and the light penetration depth in the column were measured in the water-filled column prior to the experiment using a light sensor^18^ connected to a spectrometer (USB4000, Ocean Optics, Germany). The light intensity was normalized to the value at the column surface. Spectra of wavelengths available at discrete depths are presented in the Supplementary Information.

Optodes glued to the inner wall of the column were used to non-invasively monitor the dissolved oxygen concentration in the headspace, water-saturated column, and medium reservoirs with a fiber-optic light source (FiBox, PreSens, GmbH)^16^. Two optodes were placed in the headspace, and eleven more in the saturated part of the column, between −0.2 and −18.3 cm (Fig. 1). Fe(II) and Fe(III) were monitored in aqueous samples extracted from the central vertical axis of the column, accessed through sampling ports along the side of the column (Fig. 1). Samples for iron analysis were retrieved from all six sampling ports every 1–3 days, and were quantified using the ferrozine assay^19^. Siderophore production was tested with the chrome azurol S assay^20^ after samples were centrifuged at 5000 g for 5 min.

*Synechococcus* PCC 7002 was routinely cultivated on MP medium without Fe(II)Cl_2_. Cells from a log-phase culture of *Synechococcus* PCC 7002, which had been flushed with N_2_/CO_2_ (v/v, 90/10) under light exclusion to remove oxygen, were inoculated to the medium-filled column from six ports distributed every 5 cm vertically throughout the column. The final concentration of *Synechococcus* PCC 7002 at the beginning of the experiment was 3.6 × 10^6^ cells mL^−1^. Oxygen was measured in the headspace at every 1 to 3 days. The oxygen consumption rate (negative values) and oxygen production rates (positive values) within the saturated column were calculated by fitting the measured oxygen data via PROFILE^21^, invoking both molecular diffusion and advection. Fixed boundary conditions were bottom flux and top concentration, and the oxygen diffusivity (2.2552 × 10^−5^ cm^2 ^s^−1^) at 25 °C and 30‰ salinity seawater^22^. After continuous light incubation for 20 days, the light was switched off (day 20) for two days and switched on again on day 22 in order to examine possible light-driven iron redox processes. The flow of medium was sustained during this light-dark-light incubation.

Additional batch experiments were conducted with synthetic ferrihydrite^23^ to decipher possible enzymatic and/or abiotic Fe(III) reduction processes. *Synechococcus* PCC 7002 was grown in ~40 µM Fe(II) MP medium and flushed with N_2_/CO_2_ (v/v, 90/10) for 30 min to remove oxygen_._ This culture was inoculated into serum bottles containing 50 mL anoxic medium with 1 mM ferrihydrite (8 experimental bottles, and 1 abiotic control) to a final concentration of 7.3 × 10^5^ cells mL^−1^. The bottles were incubated at a light intensity of 12.82 μM photons m^−2^ s^−1^ for 6 days, during which time cells reached 3.5 × 10^7^ cells mL^−1^ and produced oxygen. Then two (duplicate) bottles remained in the light, while two bottles were moved to a dark incubation. Cells in the other four bottles were killed by boiling for 5 min, which interrupted cell metabolic activities without destroying the cell structure^14^. Then two of the heat-killed bottles were returned to the light, and the other two were incubated in the dark. The abiotic control was kept in the light for the entire experiment. Samples were taken at intervals to measure the Fe(II) concentration. Oxygen was monitored with optodes glued into the serum bottles, as described above.

### Data Availability

The datasets generated during and/or analyzed during the current study are available from the corresponding author on reasonable request.

## Results

### Initial oxygen production and Fe(II) oxidation in the column

In order to simulate a Precambrian continental margin (in 1-dimension) from which IF deposited after ferruginous waters upwelled from a deep basin, Fe(II)-containing seawater medium was pumped into the column from ports at the bottom, and exited at the air-water interface (Fig. 1). The range of deep-water Fe(II) concentrations relevant to IF is primarily constrained by mineral saturation states in IF. Siderite saturation implies 40–120 µM Fe(II)^24^, while the presence of greenalite raises that estimate by at least one order of magnitude^25^. One estimate for upwelling of Fe(II)-containing late Archean seawater is 123 mmol m^−2^ day^−1^ (ref.^26^). Given these uncertainties in estimation of Precambrian ocean Fe(II) concentrations, a medium containing 389 µM of dissolved Fe(II) was pumped into the bottom of the column at a rate of 500 mL day^−1^, resulting in a flux of 19 mmol Fe(II) day^−1^. During initial filling of the column, Fe(II) concentrations throughout the column were in the range 60–240 μM (days 0 and 2; Supplementary Information). This drop in Fe(II) concentrations from the medium reservoir (389 µM) likely reflected initial sorption of Fe(II) to glass beads and oxidation and precipitation of some iron with residual oxygen present in the column following filling, based on similar results during an abiotic experimental column run (Supplementary Information). The abiotic experiment (described in ref.^16^ and Supplementary Information) maintained O_2_ concentrations at or below 4.7 μM, near the optimal resolution of the oxygen sensor (2.83 ± 0.14 µmol) and so 4.7 μM is our operationally-defined anoxic threshold for the column. This low level of oxygen was likely sustained through fittings, tubing, and connection, despite using the lowest oxygen diffusivity materials reasonable^16^.

**Figure 2:**
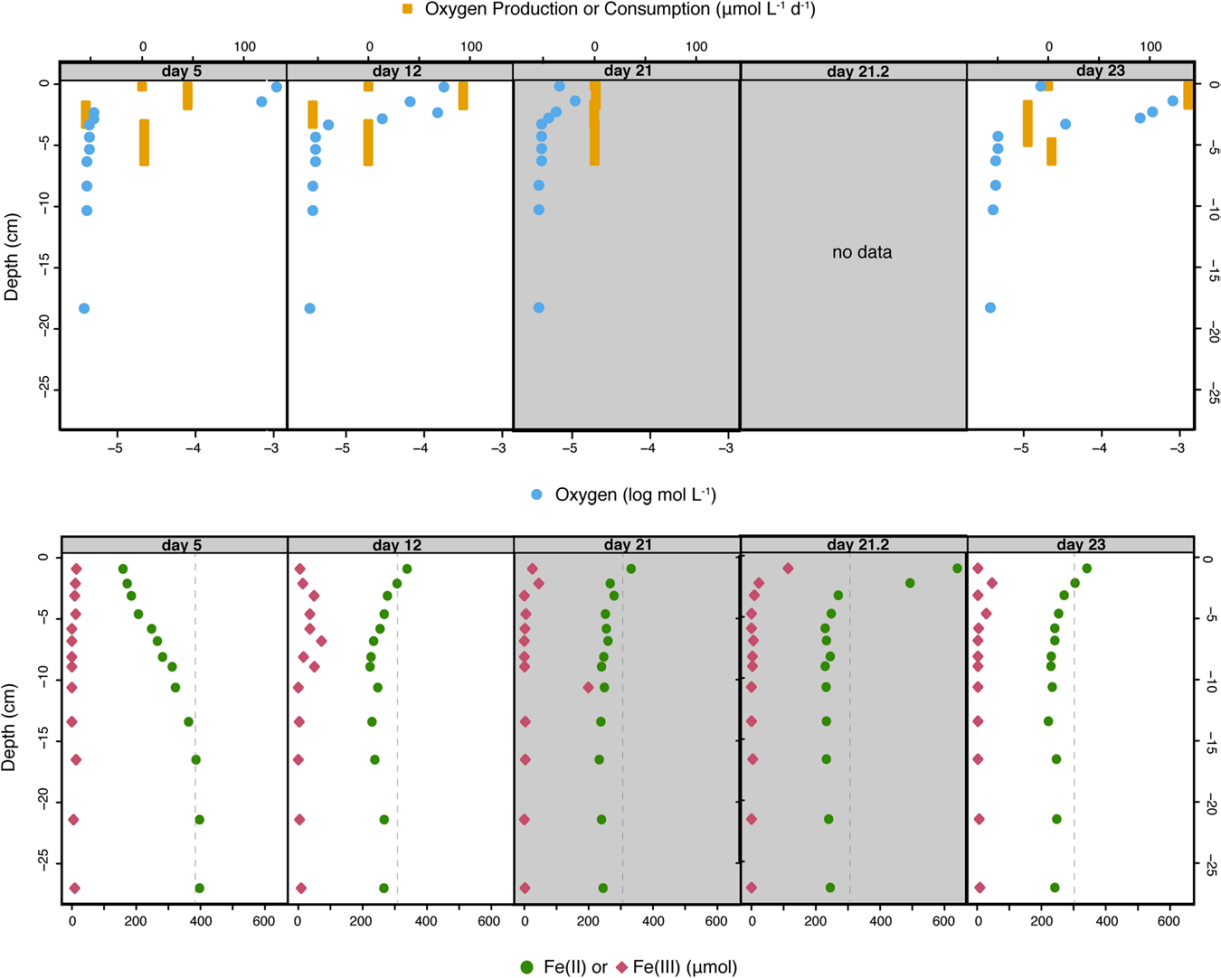
Results from the 4-week incubation of *Synechococcus* PCC 7002 in the column. Top panels show measured oxygen (blue circles) and the modeled oxygen consumption (negative) and production (positive) rates (yellow squares). Bottom panels show Fe(II) and Fe(III) concentrations measured in aqueous samples taken from the column. Dashed lines are Fe(II) concentrations of the medium reservoir. The column was incubated in the light for 20 days, then in dark on days 20 to 22 (gray panels), then in the light again for the remainder of the experiment.

After incubation of *Synechococcus* PCC 7002 for 3 days in the column, a visible accumulation of cells developed, which extended to 4 cm below (−4 cm) the top surface at the end of incubation after 34 days, where light intensity was attenuated to about 10% of that at the surface (Supplementary Information). The oxygen concentration in the headspace (3 and 4.5 cm above the water surface) remained below 11.25 μM during the 34-day experiment due to continuous flushing with N_2_/CO_2_ (v/v, 90/10). On day 5, oxygen at −0.2 cm was 1098.1 μM (Fig. 2), which exceeds oxygen saturation, and likely reflected initial proliferation of *Synechococcus* PCC 7002. A net oxygen production (44.8 μmol L^−1^ d^−1^) zone on day 5 was present from −0.2 to −1.7 cm (Fig. 2). Below −4 cm, the oxygen levels were less than 4.7 μM. The Fe(II) concentrations produced a vertical gradient by day 5, with the lowest concentrations at the top of the column. At that time, Fe(II) concentrations were 386 µM (−16.5 cm), representative of the medium reservoir (389 µM) and decreased to 159 µM at the top (−0.9 cm) of the column (Fig. 2). A net oxygen consumption zone (−55.5 μmol L^−1^ d^−1^) occurred from −1.7 to −6.3 cm, consistent with oxygen removal by oxidation of Fe(II), and the decreasing Fe(II) concentrations in this zone.

The medium reservoir was changed on day 8, and the new reservoir had an Fe(II) concentration of 307 μM (a flux of 15 mmol Fe(II) day^−1^). On day 12, the Fe(II) concentration in the bottom of the column (266 µM at −27 cm; Fig. 2) was reflective of this new input, with some loss of Fe(II) due to abiotic oxidation with oxygen that diffused into the medium reservoir and column, such as was observed in an abiotic column experiment^16^. However, day 12 Fe(II) concentrations were highest in the top of the column (338 μM at −0.9 cm). Given the pumping rate (0.5 L d^−1^) and porosity-corrected volume of the column (621 mL), the residence time of water in the column during active pumping was 1.24 days. By day 12, the volume of the column would have been replaced 3.2 times, and should reflect the Fe(II) concentration in the second medium reservoir (307 μM), or be lower due to Fe(II) oxidation. However, the elevated Fe(II) concentrations persisted, and even increased in the photic zone, reaching 453 μM at −2.1 cm on day 19, higher than in any of the medium reservoirs used in this experiment (see methods). Additionally, the oxygen concentration at day 12 had dropped, and was highest at −0.2 cm (178.8 μM). The oxygen production zone had the same extent as on day 5, but the rate was higher (e.g. 93.3 μmol L^−1^ d^−1^). Net oxygen consumption was similar to day 5 (−54.8 μmol L^−1^ d^−1^), and extended from −1.7 cm to −3.25 cm.

The Fe(III) concentration in the sampled aqueous phase was usually zero within the bottom 20 cm of the column. When Fe(III) was detectable in the extracted aqueous samples it was usually in the upper 10 cm of the column (see Supplementary Information). Fe(III) in the aqueous samples was likely Fe(III) oxyhydroxide minerals associated with *Synechococcus* PCC 7002 cells, based on microscopic observation of particles within the liquid withdrawn from sampling ports (see Supplementary Information).

### Fe(II) and oxygen profiles under light-dark-light incubation

To investigate whether the photic zone Fe(II) maximum observed by day 12 (and which persisted for the remainder of the experiment) was generated by a photochemical Fe(III) reduction process, the light was switched off on day 20. After dark incubation for 16 h, the oxygen level had decreased to a maximum of 10.9 μM at −1.4 cm on day 21 (Fig. 2). The maximum Fe(II) concentration initially dropped from 453 μM at −2.1 cm on day 19 (light on; Supplementary Figure) to 332 μM at −0.9 cm on day 21 with the light off (Fig. 2). However, it spiked nearly 5 hours later (day 21.2) to 640 μM at −0.9 (Fig. 2). On day 22, 16 hours after the light was turned on again, oxygen levels were still depressed, reaching a maximum of only 7.5 μM at −0.2 cm (see Supplementary Information), but had rebounded by day 23 to a maximum of 812.5 μM at −1.4 cm. The oxygen production rate was 137.9 μmol L^−1^d^−1^ from −0.2 to −1.7 cm (Fig. 2). The maximum oxygen values in the column remained at or above air saturation through the remainder of the experiment. Day 22 samples taken after the light was turned on had a maximum Fe(II) concentration of 357 μM at −2.1 (Fig. 2), and the maximum Fe(II) concentration in the duration of the experiment was always in the upper 3 cm of the column, and generally several hundred μM. The persistence of an Fe(II) maximum in the photic zone, despite lights being on or off, point to biological iron redox or complexation processes, in addition to any photochemical Fe(III) reduction process that may have been happening when the column was illuminated. We therefore devised an additional experiment to infer the mechanism for oxic Fe(III) reduction within the column.

**Figure 3:**
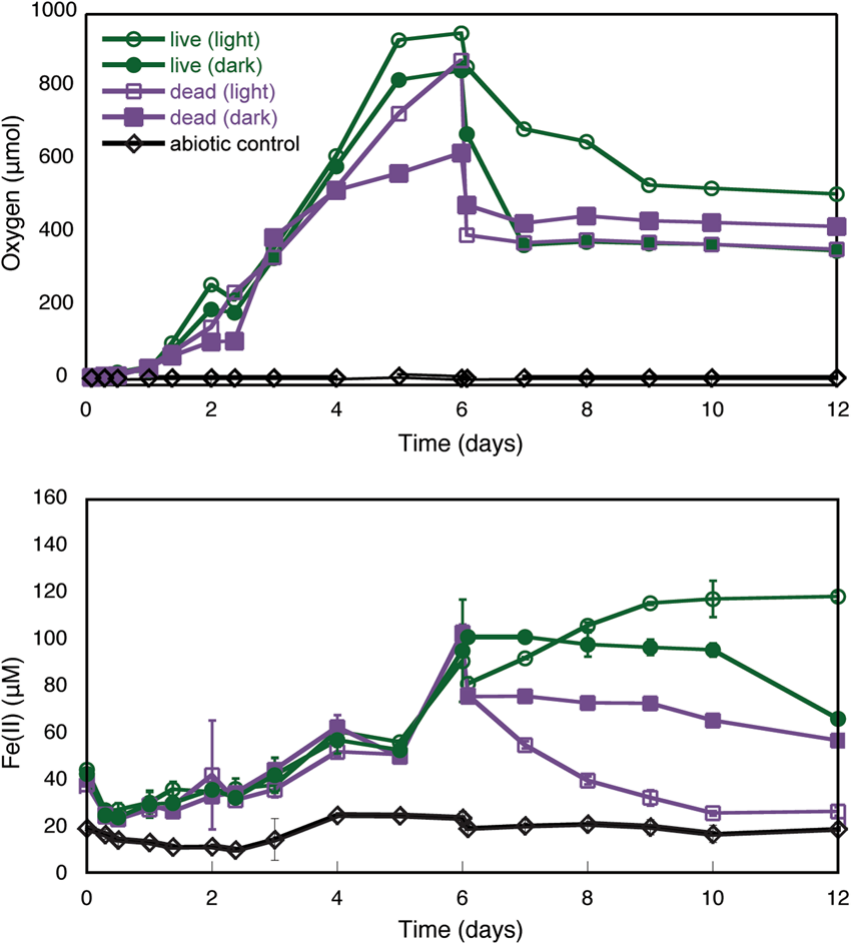
Oxygen (top panel) and Fe(II) concentrations (bottom panel) during batch incubation of *Synechococcus* PCC 7002 with ferrihydrite. All bottles were incubated in the light for 6 days, during which time cells grew and produced oxygen. Then one set of bottles (open symbols) remained in the light, while another set (closed symbols) was moved to the dark. At this time, two sets of bottles were heat-killed (squares), with a subset moved back to light incubation, and a subset incubated in the dark. Experiments were replicated, and representative results are shown for visual clarity. An abiotic control (diamonds) was constantly light incubated. Error bars show 2 standard deviations on Fe(II) measurements (analytical uncertainty). Replicate data are presented in the Supplementary Information.

Fe(III) reduction experiments were conducted with synthetic ferrihydrite, as ferrihydrite is the Fe(III) mineral formed when *Synechococcus* PCC 7002 is grown under Fe(II)-rich conditions^17^. Eight identical serum bottles containing anoxic medium with 1 mM ferrihydrite were inoculated with *Synechococcus* PCC 7002 and grown for six days in light, producing oxygen and generating Fe(II) (Fig. 3). Fe(III) reduction did not appreciably occur when cells were absent. After six days, bottles that were moved to the dark stopped producing oxygen and Fe(II) concentrations remained steady. Additional heat-killed incubations that were subsequently incubated in the light no longer produced oxygen, but Fe(II) diminished. Those that were incubated in the dark after heat-killing showed little change in Fe(II) concentrations. The decrease in oxygen concentrations in all bottles at day 6 is due to the necessity of venting the overpressure of oxygen in the bottles during the heat treatment, which brought bottles back closer to air-saturation (224 µM O_2_ for this experiment).

## Discussion

The column was designed to simulate the chemical and physical environments available at different depths along an oxygenated Precambrian continental margin subjected to upwelling of Fe(II)-rich water, from which deepwater IF were depositing (Fig. 1). While such a margin has spatial variation in the horizontal plane that our column cannot account for, the primary physical and chemical forcing we are applying to our system is upwelling/advection, which was thought to have happened along such margins^26–28^. This has been simulated through reaction modeling in one (vertical) dimension^29,30^, and our column seeks to provide a laboratory-scale analog of this^16^.

Within this context, the column simulated a habitat for planktonic marine cyanobacteria (Fig. 1). Secondary electron microscopy (SEM) of liquid extracted from the column documents the presence of cells in the liquid phase (Supplementary Information). The inclusion of glass beads was necessary to stabilize the chemical gradients developed through advection and molecular diffusion from disturbance by external perturbations (e.g. vibrations). However, the beads provided an additional benthic habitat, such is analogous to that inhabited by cyanobacteria that may have been responsible for formation of stromatolites, which are common in Archean-aged carbonate platforms^31–33^. Fluorescence microscopy documents that cells also adhered to glass beads (Supplementary Information), such as would be expected of benthic cyanobacteria^34^.

Fe(III) reduction likely occurred using Fe(III) from accumulated Fe(III) (oxyhydr)oxide minerals produced from Fe(II) oxidation. While maximally a few tens of μM Fe(III) was detectable in the aqueous samples from the column at most times, this was likely present as Fe(III) (oxyhydr)oxide minerals and Fe(III) adsorbed to the surface of *Synechococcus* PCC 7002 cells (ref.^17^ and Supplementary Information). Fe(III) (oxyhydr)oxide minerals were abundantly associated with glass beads, as determined by iron extraction following cessation of a replicated column experiment (Supplementary Information).

While Fe(II) concentrations were elevated within the photic zone from the concentrations in the reservoir during light incubation, the Fe(II) and Fe(III) concentrations in aqueous samples were observed to spike to their highest values during the dark incubation (day 21.2; Fig. 2). As this spike occurred within the colonized portion of the column in the dark, the result suggests a biological Fe(III) reduction process was occurring independent of light. For this reason, additional experiments were conducted to explore the biological and physical controls on Fe(III) reduction in the presence of *Synechococcus* PCC 7002.

The batch experiments with synthetic ferrihydrite document that *Synechococcus* PCC 7002 is capable of Fe(III) reduction. Fe(II) was produced from ferrihydrite in the presence of live cells growing in light (Fig. 3). However, heat-killed cells no longer produced additional Fe(II), regardless of whether or not they were incubated in the light or dark. This result indicates that Fe(III) reduction was a process attributable to only live cells. Furthermore, Fe(II) present when cells were heat-killed was subsequently oxidized by residual oxygen produced during initial growth. This is in contrast to light grown cells that were moved to a dark incubation, where Fe(II) produced in the light did not experience as much subsequent oxidation as in heat-killed incubations, despite similar amounts of oxygen. Production of Fe(II) from cells always incubated in light occurred until the end of the experiment, when cells were likely in stationary phase^17,30^. Our results indicate that Fe(III) reduction is attributable to live cells, and can occur under light or dark conditions.

Extracellular Fe(III) reduction has been proposed as a pathway necessary for uptake of iron as Fe(II), and such an iron acquisition pathway may be widespread among phytoplankton^35,36^. Fe(III) reduction has been observed in several cyanobacteria^13,14^, yet the reported mechanisms of Fe(III) reduction vary^36^. Therefore, several Fe(III) reduction pathways may be relevant to interpret the results of our experiment. Siderophores complex and dissolve Fe(III), and are synthesized and secreted by microorganisms under iron limitation^37^. Siderophores are widely used by marine photosynthetic bacteria as a substrate for Fe(III) reduction before uptake of Fe(II)^38^. *Synechococcus* PCC 7002 is known to secrete siderophores^39^, and other cyanobacteria do as well^37^. Siderophore production was characterized in *Synechococcus* PCC 7002 under iron-limiting growth conditions^17,37,40^. No siderophores were detected in either our column or batch experiments. This is consistent with the lack of siderophore-related proteins produced by this strain under non-limiting iron concentrations similar to the current experiments^17^. Thus, reduction of siderophore-bound Fe(III) seems unlikely to be significant in the column.

Another possible Fe(III) reduction pathway is via the extracellular production of the reactive oxygen species (ROS) superoxide. Superoxide can oxidize Fe(II), but can also reduce Fe(III), especially if Fe(III) is in aqueous or ligand-bound form^11^. Many marine algae^41^ and a wide range of marine bacteria^42^ are capable of producing superoxide, suggesting marine superoxide cycling can occur independent of light. The coastal marine cyanobacterium *Lyngbya majuscule* generates superoxide for Fe(III) reduction and Fe(II) uptake^14^. NAD(P)H oxidoreductases are known or thought to be responsible for superoxide production in many biological systems^14,42,43^, as superoxide production can be inhibited by the addition of diphenyleneiodonium (DPI), which is known to act on these enzymes^23,40^. Addition of DPI to incubations of *Synechococcus* PCC 7002 completely inhibited growth (Supplementary Information), indicating that NAD(P)H oxidoreductases are also present and essential to the growth of this strain. Furthermore, Fe(III) is known to bind to the surface of *Synechococcus* PCC 7002 cells under similar conditions as within the column, perhaps to a capsular exopolysaccharide^17^, which could make it more susceptible to superoxide-mediated reduction^11^. Therefore, superoxide production by *Synechococcus* PCC 7002 seems to be a plausible light-independent pathway for Fe(III) reduction within the column.

Fe(III) reductases have also been suggested as an alternative mechanism for diverse planktonic cyanobacteria to reduce Fe(III) before uptake^13,44^. In one molecular model for *Synechocystis* 6803, Fe(III) is transported through the outer membrane, reduced to Fe(II) in the periplasmic space, and then transported into the cell^45^. When *Synechococcus* PCC 7002 is grown in Fe(II)-rich conditions, similar to the conditions within the column, Fe(III) is observed at the surface of the cell, and a number of iron uptake and receptor proteins were abundant^17^. These observations suggest that this strain may actively bind iron at its surface, which could make it subject to active reduction as well.

Finally, photochemical reduction may also enhance the biologically mediated Fe(III)-reducing activity we observed. Ligand-bound Fe(III) is reduced to Fe(II) and released in LMCT, which has been shown to promote Fe(II) uptake in the freshwater cyanobacterium *Microcystis aeruginosa*^46^. Furthermore, photochemical reduction of Fe(III) bound to ligands, including siderophores, is common in the open ocean^47^. Generally, these processes are thought to occur with UV light^47^, but up to 30% of photochemical Fe(III) reduction may be attributed to visible wavelengths^48^. Our light spectrum profile of the column indicates that visible wavelengths are still abundant at 2 cm below the surface (Supplementary Information), within the zone of Fe(II) production (Fig. 2). Furthermore, Fe(II) concentrations in the dark incubation of live cells stayed relatively constant until the end of the experiment, when cells may have died, consistent with continued biological Fe(II) production even in the dark. Therefore, we cannot rule out that photochemical processes might have also contributed to the Fe(III) reduction occurring in the column.

Our laboratory simulation shows that cyanobacterial enzymatic reactions can reduce Fe(III) in the presence of free oxygen. In our experiments, oxygen values ranged from well above air saturation (224 μM in this experiment) down to a few μM when and where Fe(III) reduction occurred (Figs 2 and 3), indicating that oxic Fe(III) reduction is possible under a wide range of oxygen concentrations. Oxygen concentrations in the late Archean ocean may have reached 1–10 μM^49^, or even up to 35 μM locally^29^. Surface values below 5 μM are suggested into the Proterozoic^50^, while in the Neoproterozoic, poorly oxygenated zones (ca. 10 μM) may have persisted at interfaces with upwelling Fe(II)-rich water below more oxygenated conditions^51^. Furthermore, oxygen minimum zones with tens of μM oxygen were present in the Proterozoic^52^, which is characterized by ferruginous conditions throughout much of the oceans^1–4^. The expansion of oxygen minimum zones in the modern ocean^53^ raises the possibility that cyanobacterial-driven Fe(III) reduction may be relevant in deeper waters, as deep chlorophyll maxima are sometime observed at the boundary to more nutrient-rich and often oxygen-depleted deep waters^54^. Thus, our results are relevant to mildly to fully oxygenated interfaces with ferruginous waters throughout Earth’s history, following the possible appearance of oxygen in the surface oceans as early as 3 Gy ago^55^.

While a convergence of evidence documents the importance of light dependent Fe(III) reduction in modern oxic seawater conditions^8,38,48,56^, our work indicates that this pathway, in combination with Fe(III) reduction at cyanobacterial surfaces^13^, is also significant when and where iron concentrations are much higher (e.g. hundreds of micromolar) than those in the modern surface ocean (pico to nanomolar range). As our experiments involved growth of cyanobacteria, and due to varying numbers of cells, the oxic Fe(III) reduction we observed was unlikely to be in steady-state with abiotic Fe(II) oxidation. Therefore, it was impossible to parse the quantitative impact of oxic Fe(III) reduction on iron turnover. However, prior estimates for Fe(III) reduction in the presence of cyanobacteria allow for us to make an estimate. A rate of 115 × 10^−21^ mol cell^−1^ hr^−1^ of Fe(III) reduced in light, which integrates abiotic photochemical Fe(III) reduction, was measured for *Synechocystis* 6803^13^. Given a concentration of cyanobacteria in the surface ocean of ca. 10^5^ cells mL^−1^ (ref.^57^), the combination of these processes could reduce Fe(III) at a rate of 1.15 × 10^−11^ mol hr^−1^. Oxidation rates for Fe(II) are extremely sensitive to oxygen and iron concentrations, as well as pH and temperature. Using a half-life for similar pH and Fe(II) concentration (ca. 200 nM) of 1413 min^−1^, a comparable Fe(II) oxidation rate is 1.86 × 10^−9^ mol hr^−1^ (ref.^58^)_._ These calculations predict that for oxic Fe(III) reduction to be effective in maintaining Fe(II) in oxic waters, cells would need to be quite dense, such as might occur within a mat, a bloom, or a deep chlorophyll maximum. However, we note that comparable rates for the higher Fe(II) concentrations we utilized are lacking. Our empirical observations of the surface maximum of Fe(II) (Fig. 2) suggest that oxic Fe(III) reduction can outpace Fe(II) oxidation in the conditions we tested.

While Fe(III) reduction is thought to be a mechanism used by cyanobacteria for iron uptake in iron-limiting conditions^13,14^, which characterize many modern aquatic habitats, it also appears to be a widespread phenomenon in algae^36,41^. This likely stems from the large demand for iron in the photosynthetic machinery^59^, which may harken back to the iron-replete Precambrian oceans from which many oxygenic photosynthetic organisms evolved. Deep hydrothermally-sourced Fe(II), supplied via upwelling to some shallow-water settings^27,28,31^, would have been oxidized with photosynthetic oxygen, thus titrating iron out of solution^60^. Therefore, an Fe(III) reduction strategy might have been necessary for even the earliest cyanobacteria to acquire iron as they modified the redox potential, and thus availability of iron in their environment.

Clearly, oxic Fe(III) reduction could have played a distinct role in Precambrian iron cycling. Yet rapid iron redox cycling has been invoked primarily within the interface of oxic to anoxic sediments or waters (e.g. refs^31,61,62^), mediated either by dissimilatory Fe(III)-reducing microbes or reductive dissolution of Fe(III) (oxyhydr)oxide minerals by hydrogen sulfide. There are several examples where oxic Fe(III) reduction may help to explain conflicting interpretations of oxygen levels based on different redox proxies. The 2.5 Gy old Campbellrand-Malmani Platform in South Africa preserves subtidal to supratidal depositional settings^63^, and contains encrustations of decimeter to meter-thick bedding of aragonite and calcite crystals, while lacking significant micrite^9,12^. This has been attributed to Fe(II) inhibiting the crystal growth of calcite and aragonite, as Fe(II) suppresses new crystal nucleation rates^64–67^. Yet the geochemistry of the succession suggests it was an oxygen oasis^29,33,68–70^. Iron and molybdenum isotope systematics indicate the presence of Fe(II) in shallow seawater^29,60^, and detailed mineralogical investigation indicates that iron was incorporated into shallow-water carbonate minerals as Fe(II) from coeval seawater^60^. The conflicting evidence for Fe(II) in seawater in the presence of oxygen could be reconciled by invoking oxic Fe(III) reduction by organisms similar to modern cyanobacteria. Importantly, there were extensive microbial mats, recorded as stromatolites from the section, and photosynthetic lifestyles cannot be ruled out^33^. Even earlier in the Archean, deposition of iron-bearing stromatolites from the ca. 2.8 Gy old Steep Rock carbonate platform also indicate periodic incursion of Fe(II)-rich seawater into an oxygen oasis, signified by the deposition of iron-poor limestone and rare earth element patterns consistent with oxygen^31,71^. These stromatolites also have features parsimonious with the presence of cyanobacteria-like organisms^71^, highlighting a role for oxic Fe(III) reduction at such settings.

Our work extends the implications that Fe(III) reduction can occur under oxic conditions of modern marine systems to Precambrian marine settings that are typified by ferruginous deep seawater overlain by oxygenated seawater. Oxic Fe(III) reduction, mediated enzymatically and perhaps augmented by photochemistry, can persistently generate Fe(II) from Fe(III) (oxyhydr)oxides and/or ligand-bound Fe(III), even from Fe(II)-rich water masses. As redox interfaces between deep, Fe(II)-rich seawater and oxygenated seawater persisted for billions of years, oxic Fe(III) reduction may have played an important role in marine iron cycling for as long.

## Electronic supplementary material

Supplementary Information
