# Supplementary material for: Oxic Fe(III) reduction could have generated Fe(II) in the photic zone of Precambrian seawater
Source: Sci Rep. 2018 Mar 9;8:4238. doi: 10.1038/s41598-018-22694-y (PMC5845006; doi:10.1038/s41598-018-22694-y)

SUPPLMENTARY INFORMATION

Oxic Fe(III) reduction could have generated Fe(II) in the photic zone of Precambrian seawater

Elizabeth D. Swanner[1,2]*, Markus Maisch[2], Wenfang Wu[2,3], and Andreas Kappler[2]

[1]*Department of Geological and Atmospheric Sciences, Iowa State University, Ames, IA, United States of America*
[2]*Department of Geosciences, University of Tuebingen, Tuebingen, Germany*
[3]*Key Laboratory of Earth and Planetary Physics, Institute of Geology and Geophysics, Chinese Academy of Sciences, Beijing, China*
**Correspondence should be addressed to E.D.S. (email: eswanner@iastate.edu)*

Additional column experiments

The complete dataset of oxygen, Fe(II) and Fe(III) values for the experiment described in Figure 1 is available as Supplementary Figures 1 and 2.

An abiotic control experiment was performed in the column using a medium reservoir with 425 µM Fe(II). The results of this experiment have been previously described[1]. A total volume of 5 L was pumped through the column over 10 days. The MP medium pump rate flow of 0.45 L day^{-1} resulted in a flux of 19 mmol Fe(II) m^{-2} d^{-1}. Supplementary Figure 3 shows how column Fe(II) concentration responds to the during filling. Note that lower concentrations at the top of the column on day 1 result from sorption of Fe(II) and/or oxidation of Fe(II) and precipitation of Fe(III) (oxyhydr)oxides onto glass beads (see below).

A duplicate biotic experiment was performed with *Synechococcus* PCC 7002 for 20 days. Data from the first seven days of this duplicate experiment have been previously published[1], and are used here with permission from the publisher. The Fe(II) concentration in the medium reservoirs was 320 µM. The resulting oxygen, Fe(II) and Fe(III) profiles from all time points sampled over 20 days are presented as Supplementary Figures 4 and 5.

Additional batch experiments

The batch experiments presented as Figure 3 in text had duplicates for each experimental condition. For visual clarity, only representative results were plotted. All experiments are presented in Supplementary Figure 6.

Synechococcus PCC 7002 cells were inoculated in triplicate into two sets of 100 ml MP medium in buty rubber-stoppered glass bottles. The medium contained 0.5 mM Fe(II)Cl_2. One set of three bottles contained 50 µM diphenyleneiodonium (DPI), which inhibits NAD(P)H oxidoreductases. Fe(II) oxidation was followed during the 72 h light incubation with Ferrozine, while growth was assessed visually at the end of the experiment. Fe(II) was oxidized in cultures without DPI (Supplementary Figure 7), due to the production of oxygen[2]. In cultures with DPI, growth was arrested, as evidenced by a photograph taken at the end of the 72 h incubation.

Light characterization

As the column is sealed during experiments, it was not possible to quantify the *in situ* light intensity. Therefore, the light intensity with depth was characterized in a glass cylinder with identical dimensions, the same glass beads, the same liquid medium, and the same light source. A light sensitive microsensor (Unisense A/S, Aarhus, Denmark) [3] connected to a spectrometer (USB4000, Ocean Optics, Germany) was placed several cm deep within the column and covered with glass beads, then the column was filled with medium. Stray light was attenuated by covering the cylinder with opaque black tape and black cloth. The light source was positioned over the cylinder. The light intensity for each depth was calculated by integrating the detected intensities (microsensor calibration: 200, 3 to average) per wavelength within the photosynthetically active light spectra range covering wavelengths from 400 - 710 nm[4]. The measured light intensity at each depth was recorded and normalized to the initial light intensity in air within the headspace of the column. Wavelengths present at different depths in the column are shown in Supplementary Figure 8.

Areas within water columns having a minimum of 10% light irradiance from the surface can be described as the photic zone [5]. Areas above 3.8 cm depth within the water-filled column used in this study have a relative light intensity (RLI) ≥10% (Supplementary Figure 9). A RLI of 10% in the current study comprise a photosynthetic photon flux (PPF) of 27 µmol m^{-2} s^{-1} [6], which should promote the growth of the genus *Synechococcus* [7, 8]. The oxygen concentration profiles and zones of production or consumption in Figure 2 delineate that the upper 2.5 cm of the column was likely the zone of photosynthetic growth in the present experiment.

Iron sorption and iron phases in column experiment

After the abiotic column experiment, 10 g of glass beads were collected and soaked with 6N HCl. The amount of iron sorbed or precipitated onto the glass beads was quantified from triplicate extractions, and had an average iron content of 4.06 ± 0.95 µg g^{-1} of glass beads (1 standard deviation).

In order to quantify the iron sorption and iron precipitation onto glass beads in the top of the biotic column experiment, a push core of the glass beads was collected from the upper 6 cm of the replicated biotic column using a plastic 60 ml syringe with the tip cut off. The syringe was inserted into the glass beads as the plunger was gently pulled. After the syringe was filled the open end was capped with another plunger and the sample was transported into an anoxic glovebox. The beads were separated into samples by intervals of 0.5 cm, so that a depth reported as -0.5 cm is bulk data from 0 to -0.5 cm.

Iron was sequentially extracted in a procedure modified from ref. [9]. The beads were initially washed with anoxic ultrapure water and anoxic 1 M HCl. Then, sorbed iron (Fe_{sorb}) was extracted with anoxic 0.5 M sodium acetate (pH 4.85), incubated for 24 h with the glass beads. The glass beads were then vortexed for 5 s and the liquid centrifuged (16,000 g, 5 min). Finally, anoxic 6N HCl was used to dissolve the precipitated iron (Fe_{ppt}). Iron in the extracts was quantified using the Ferrozine assay. The concentration of Fe in the extracted solutions were converted to mass of accumulated iron using the porosity (Φ = 0.379) and assuming precipitates had the chemical formula $Fe(OH)_3$.

Supplementary Figures 10 shows the amount of Fe_{sorb} and Fe_{ppt} formed at each depth. The area around -1.5 to -2 cm shows the greatest accumulation of iron. This is similar to the depths at which maximum Fe(II) concentrations were observed (Figure 2 and Supplementary Figures 2 and 5.)

SEM and fluorescence microscopy

A Leica DM5500B microscope optical and fluorescent was used to visualize particles and bacteria within the column. Samples from the replicated biotic column experiment were collected on day 15 from a depth of 2.1 cm. Liquid samples were fixed with paraformaldehyde (PFA) at a final concentration of 2.5% and stored at 4°C until analysis. In order to investigate the allocation of cyanobacteria and to define the distribution of cells within the mineral precipitates, overlays of bright field and Cy3 filtered (used to visualize autofluorescent *Synechococcus* PCC 7002) images collected with a 40x objective were constructed using the Leica Software ver. 3.7.10. Samples from glass beads that were collected at the end of the experiment from the top 1 cm were affixed to a glass microscope slide using MP stock medium.

For scanning electron microscopy (SEM), a liquid sample was taken from the photic zone of the column on day 14. The sample was centrifuged at 7,000 g for 10 min, liquid removed, and the pellet was resuspended in 50 µL MP medium. One drop was then loaded onto a sticky carbon pad (Plano GmbH, Wetzlar, Germany) and allowed to adhere for 5 - 10 min. The carbon pad was then gently washed three times with ultrapure water in order to remove salt crystals. The carbon pad was dried in a vacuum chamber (-0.9 bar) for 3 hours, mounted onto an aluminum stub (Plano GmbH, Wetzlar

Germany) and sputter-coated with 6 - 9 mm gold (BAL-TEC SCD 005, BALTEC, Liechtenstein, 35 nm working distance, 30 mA, 60 s). SEM imaging was performed on a Leo Model 1450VP SEM (Carl Zeiss SMT AG, Germany), operated at 7 kV with a 7 mm working distance.

Images collected by fluorescence and secondary electron microscopy are shown in Supplementary Figure 11. The fluorescence image shows *Synechococcus* PCC 7002 cells adhered to the surface of glass beads. This provides evidence that the column also simulated a benthic setting, where cyanobacteria grow in on sediment grains[10]. The SEM image shows that cells in the aqueous samples were associated with minerals – likely Fe(III) (oxyhydr)oxides. This likely accounts for the Fe(III) detected from aqueous samples (Figure 2).

Supplementary References

1. Maisch M, Wu W, Kappler A, Swanner ED. Laboratory Simulation of an Iron(II)-rich Precambrian Marine Upwelling System to Explore the Growth of Photosynthetic Bacteria. *Journal of Visualized Experiments* **113**, e54251 (2016).

2. Swanner ED*, et al.* Physiology, Fe(II) oxidation, and Fe mineral formation by a marine planktonic cyanobacterium grown under ferruginous conditions. *Frontiers in Earth Science* **3**, (2015).

3. KÜhl M, JØrgensen BB. Spectral light measurements in microbenthic phototrophic communities with a fiber-optic microprobe coupled to a sensitive diode array detector. *Limnology and Oceanography* **37**, 1813-1823 (1992).

4. Manodori A, Melis A. Cyanobacterial Acclimation to Photosystem I or Photosystem-II Light. *Plant Physiology* **82**, 185-189 (1986).

5. Herring PJ. *Light and Life in the Sea*. Cambridge University Press (1990).

6. Thimijan RW, Heins RD. Photometric, Radiometric, and Quantum Light Units of Measure - a Review of Procedures for Interconversion. *Hortscience* **18**, 818-822 (1983).

7. Nowack S*, et al.* The molecular dimension of microbial species: 2. Synechococcus strains representative of putative ecotypes inhabiting different depths in the Mushroom Spring microbial mat exhibit different adaptive and acclimative responses to light. *Frontiers in Microbiology* **6**, (2015).

8. Klotz A, Reinhold S, Doello S, Forchhammer K. Nitrogen starvation acclimation in *Synechococcus elongatus*: redox-control and the role of nitrate reduction as an electron sink. *Life* **5**, 888-904 (2015).

9. Swanner ED*, et al.* Iron Isotope Fractionation during Fe(II) Oxidation Mediated by the Oxygen-Producing Marine Cyanobacterium Synechococcus PCC 7002. *Environmental Science & Technology* **51**, 4897-4906 (2017).

10. Noffke N, Gerdes G, Klenke T. Benthic cyanobacteria and their influence on the sedimentary dynamics of peritidal depositional systems (siliciclastic, evaporitic salty, and evaporitic carbonatic). *Earth-Science Reviews* **62**, 163-176 (2003).

Supplementary Figure 1. Oxygen profiles for the column experiment described in the main text.

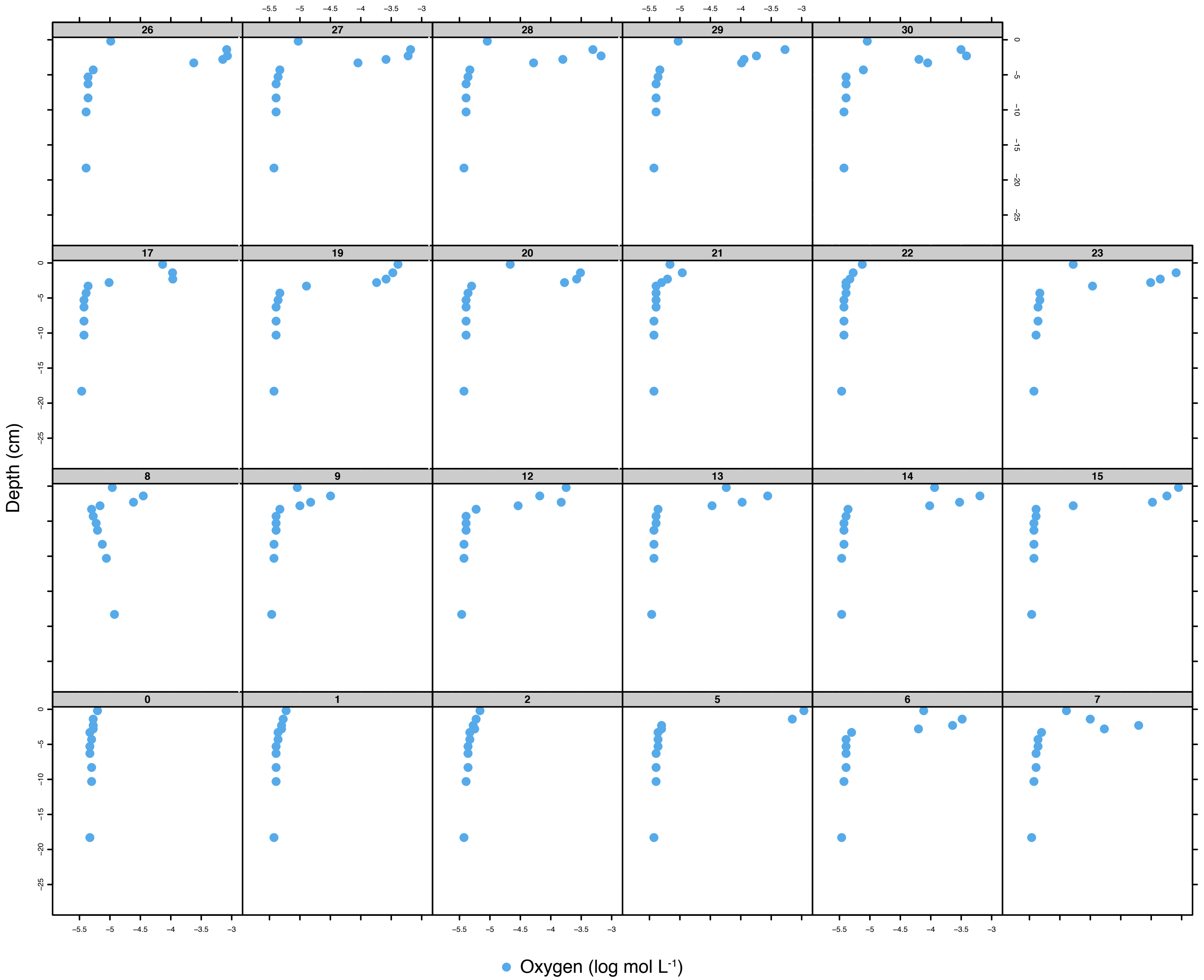

Supplementary Figure 2. Fe(II) and Fe(III) profiles from a the column experiment described in the main text.

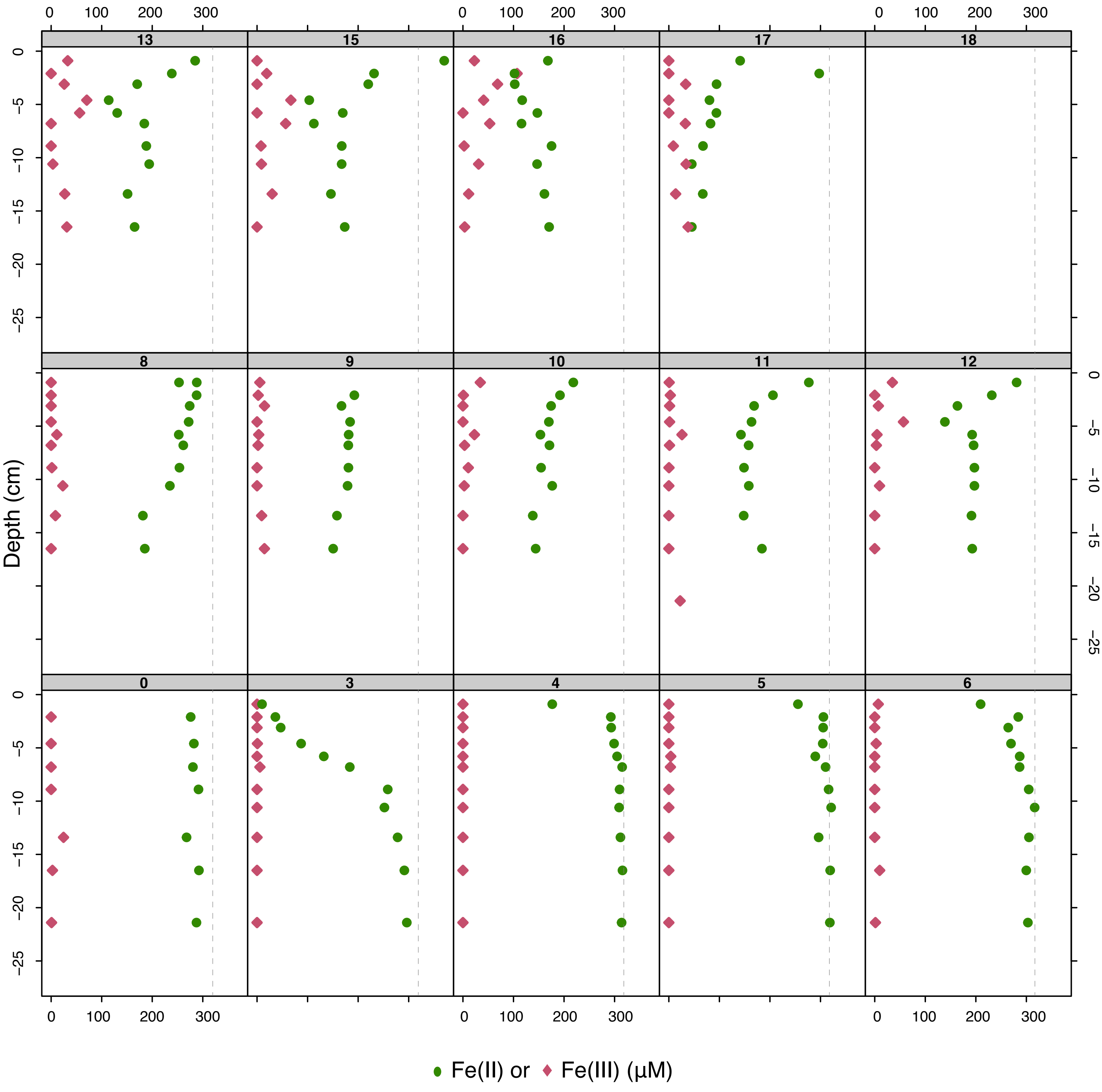

Supplementary Figure 3. Fe(II) and Fe(III) profiles from an abiotic column experiment.

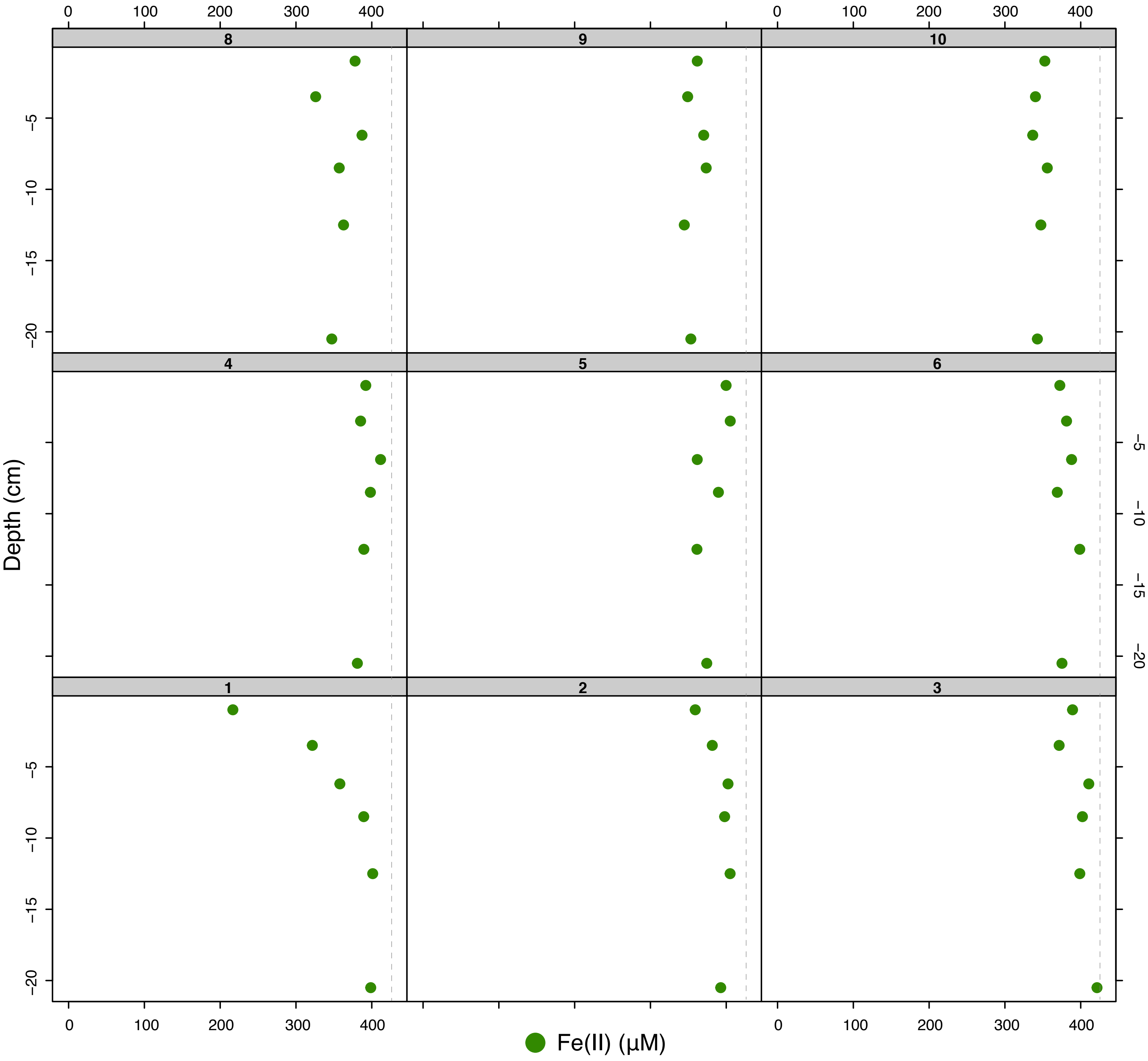

Supplementary Figure 4. Oxygen profiles from a replicated column experiment analogous to the one described in the main text. Some data from this figure have been previously published in Maisch, M.; Wu, W.; Kappler, A.; Swanner, E. D., Laboratory Simulation of an Iron(II)-rich Precambrian Marine Upwelling System to Explore the Growth of Photosynthetic Bacteria. Journal of Visualized Experiments 2016, 113, (113), e54251, and are replotted here with permission of the publisher.

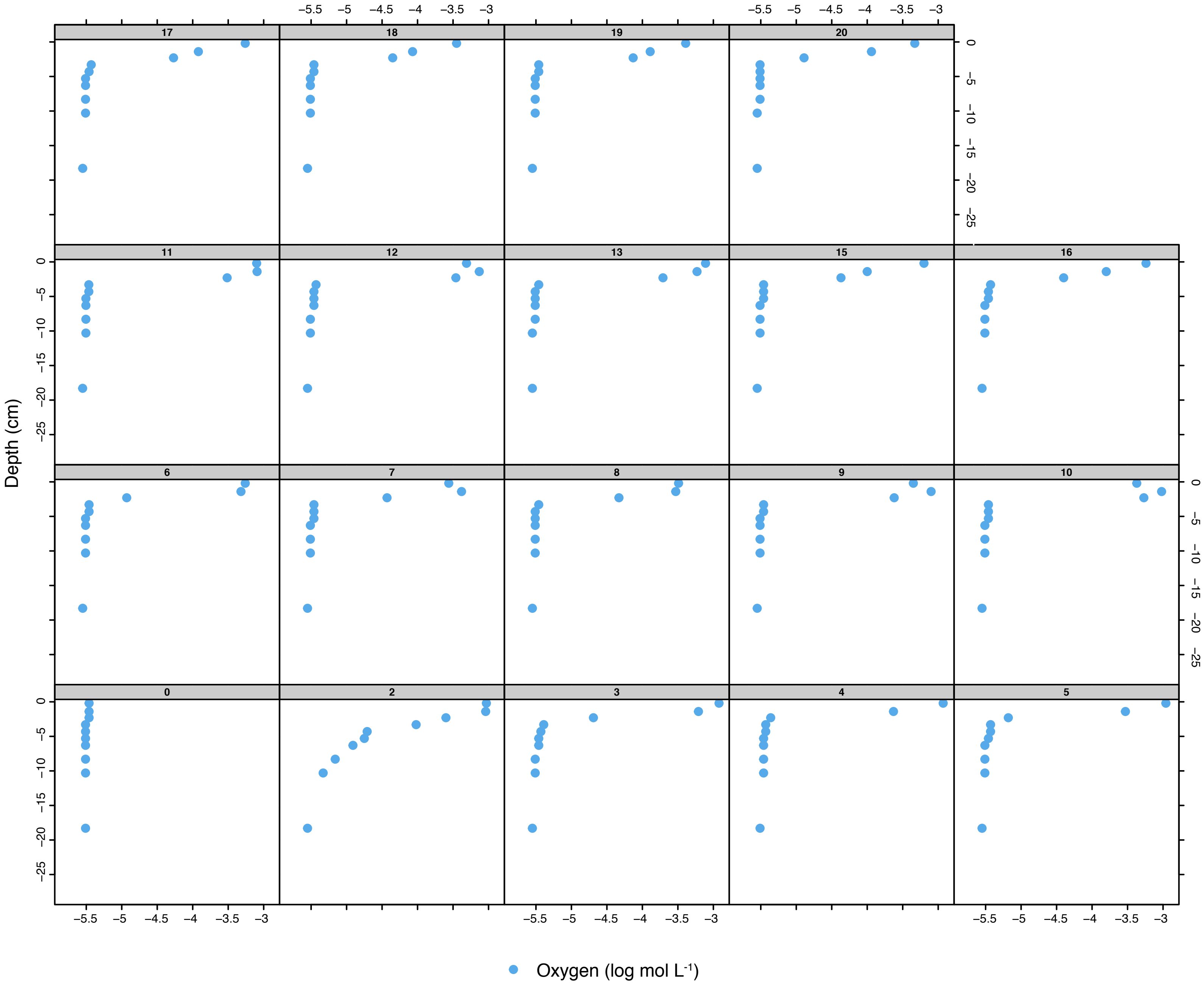

Supplementary Figure 5. Fe(II) and Fe(III) profiles from a replicated column experiment analogous to the one described in the main text. Some data from this figure have been previously published in Maisch, M.; Wu, W.; Kappler, A.; Swanner, E. D., Laboratory Simulation of an Iron(II)-rich Precambrian Marine Upwelling System to Explore the Growth of Photosynthetic Bacteria. Journal of Visualized Experiments 2016, 113, (113), e54251, and are replotted here with permission of the publisher.

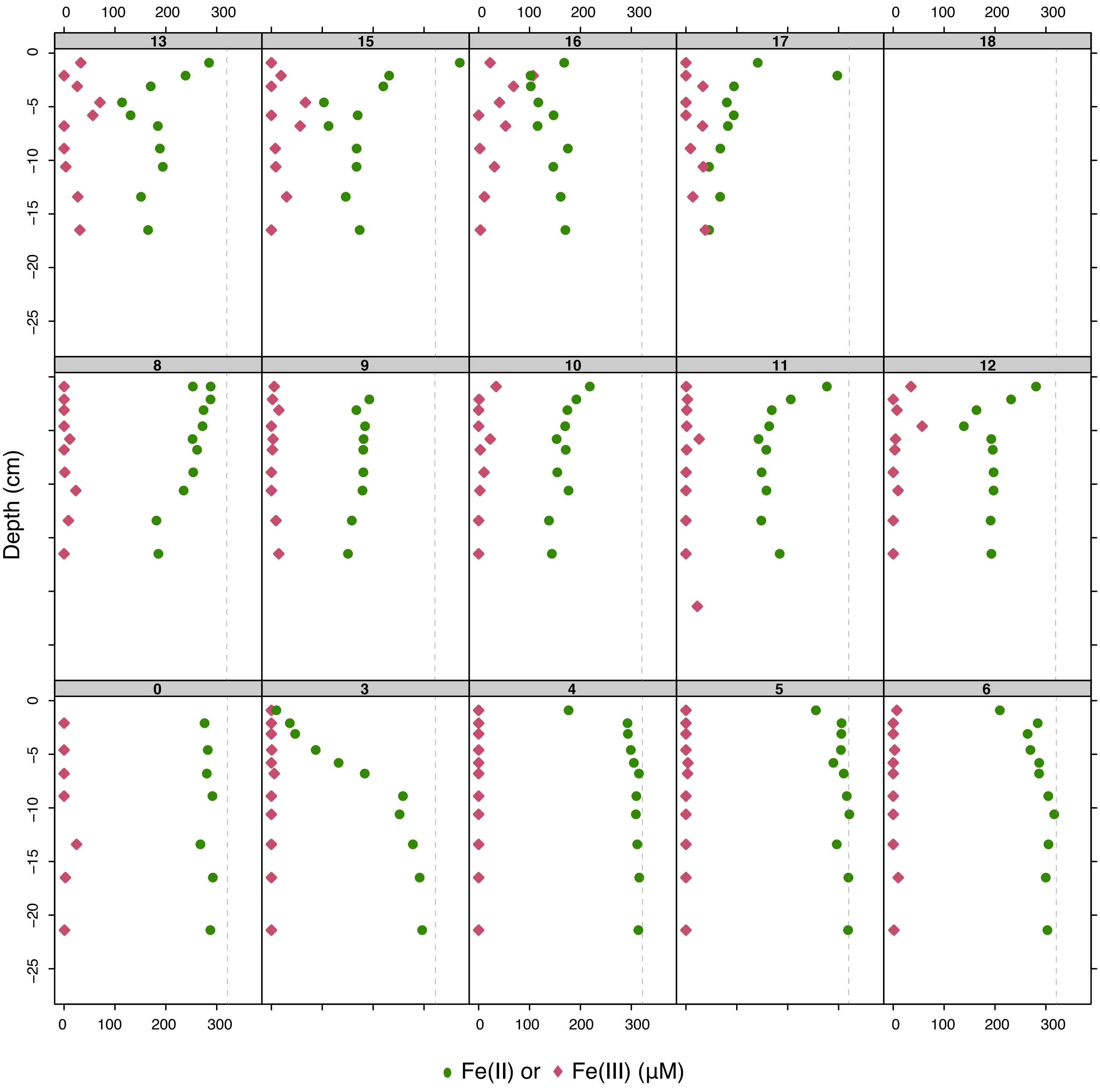

Supplementary Figure 6. Oxygen (top panel) and Fe(II) concentrations (bottom panel) during batch incubation of Synechococcus PCC 7002 with ferrihydrite. All bottles were incubated in the light for 6 days, during which time cells grew and produced oxygen. Then one set of bottles (open symbols) remained in the light, while another set (closed symbols) was moved to the dark. At this time, two sets of bottles were heat-killed (squares), with a subset moved back to light incubation, and a subset incubated in the dark. Duplicate experiments are shown. An abiotic control (diamonds) was constantly light incubated. Error bars show 2 standard deviations on Fe(II) measurements (analytical uncertainty).

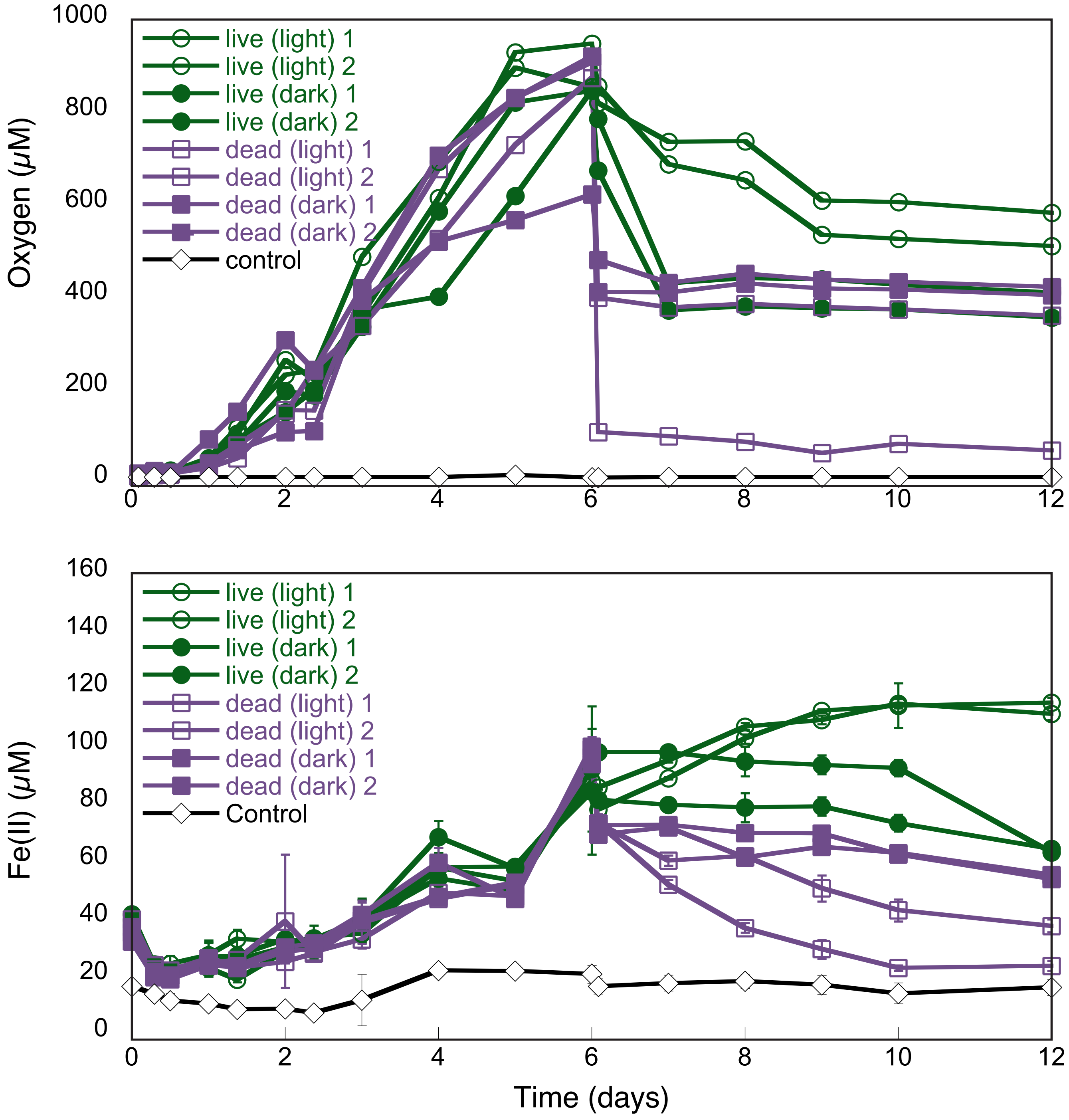

Supplementary Figure 7. Top shows one replicate each from incubations without (left) or with (right) 50 μM DPI. Plots below correspond to the Fe(II) concentration tracked in triplicates of incubations without (left) or with (right) 50 μM DPI.

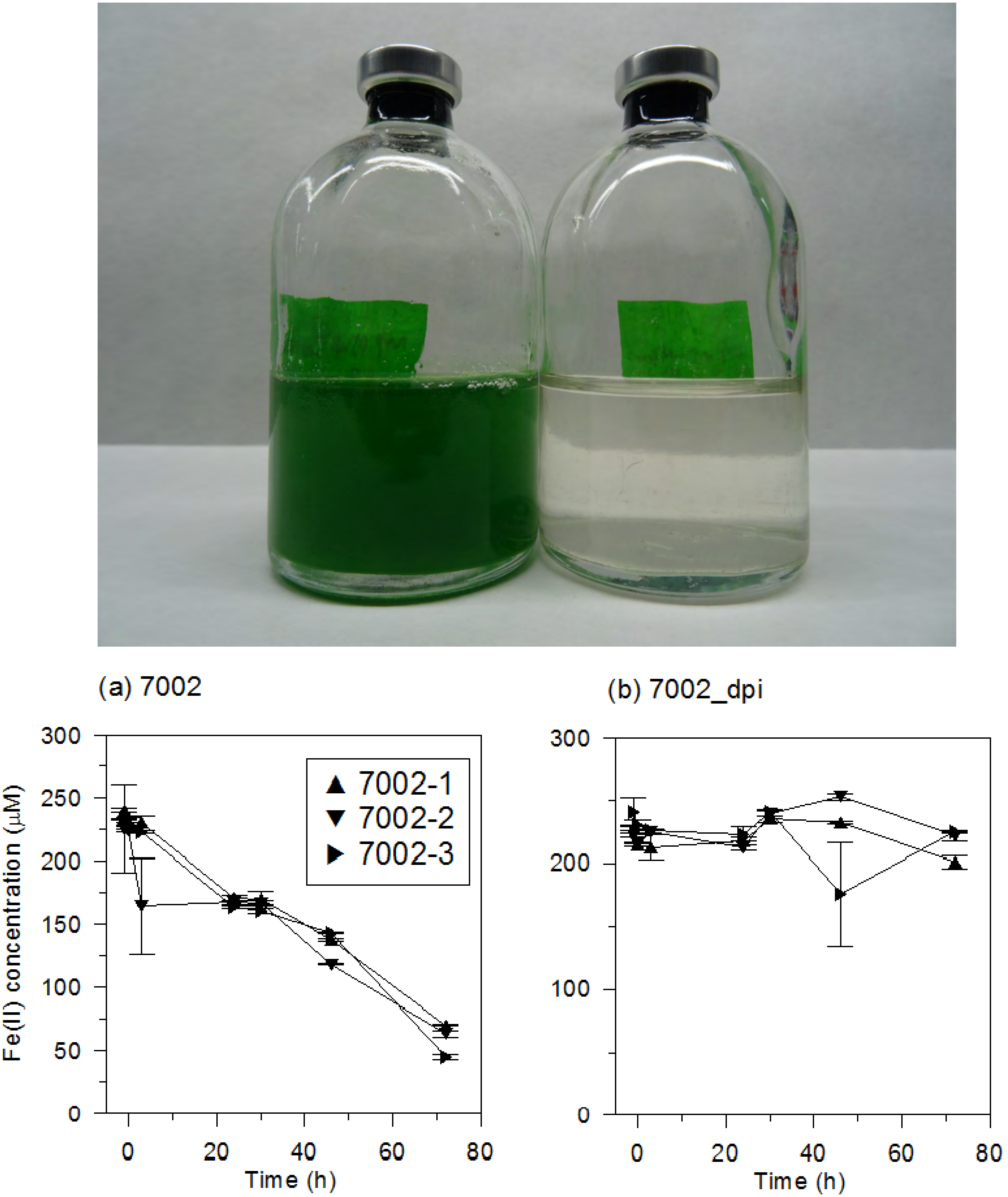

Supplementary Figure 8. Wavelengths of visible light (400-700 nm) available at different depths within the column.

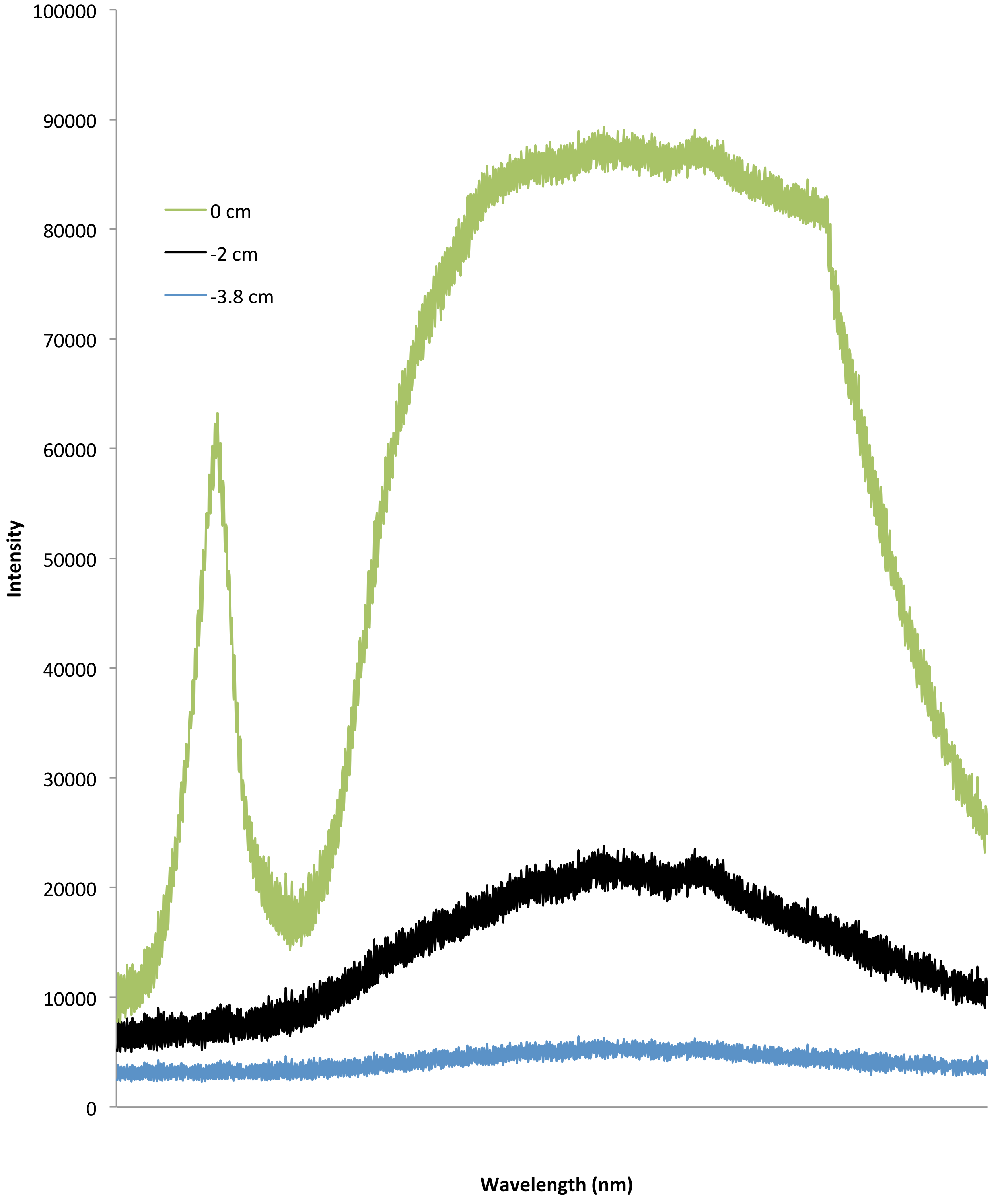

Supplementary Figure 9. Relative light intensity (RLI, in %) with depth in a water-filled column. Grey x symbols are measurements, and the fitted exponential trendline has the equation: RLD(d) = 82.253$e^{-0.94d}$ (R^2 = 0.9747).

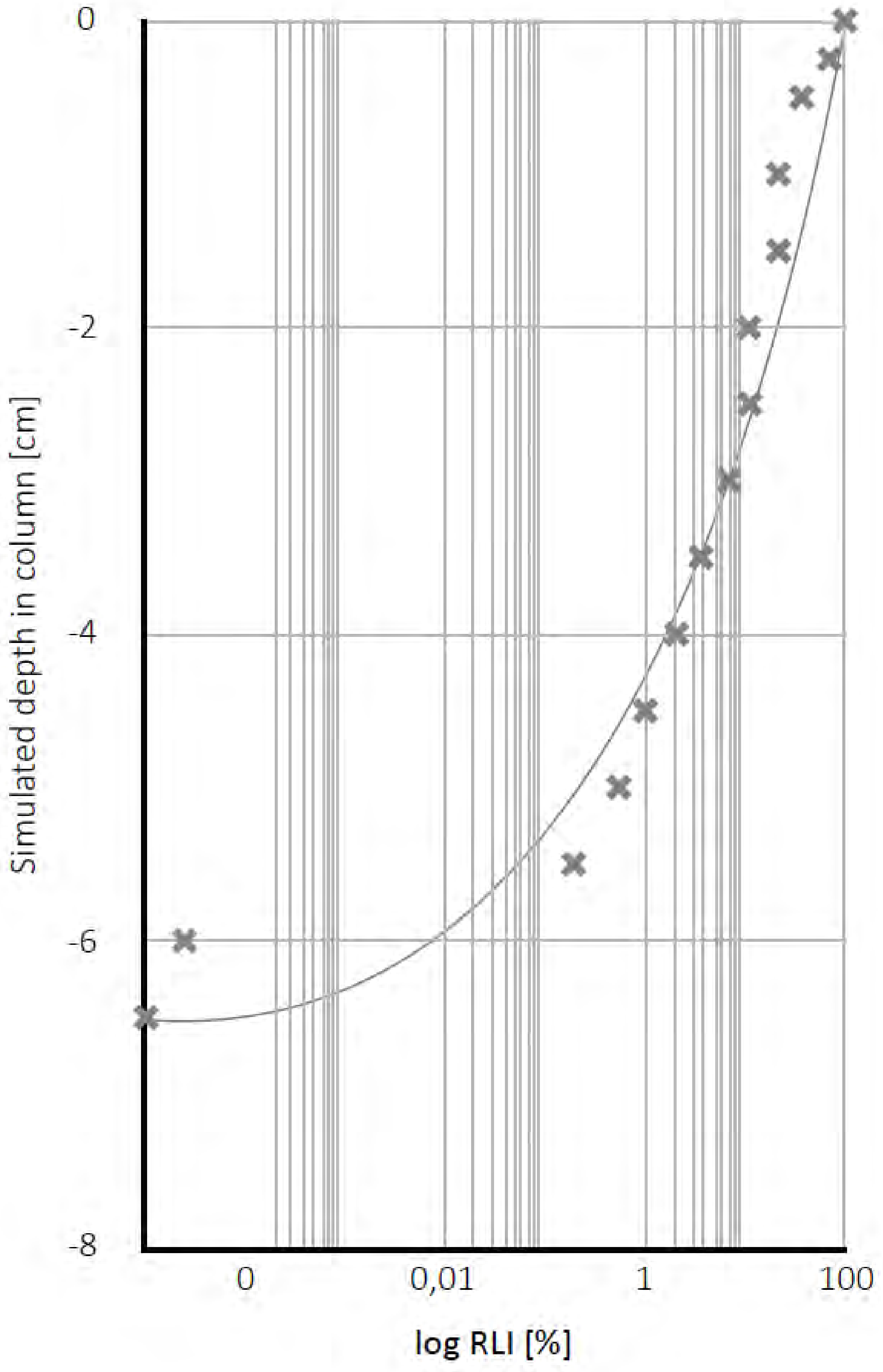

Supplementary Figure 10. Results of iron extractions from glass beads, showing the zone of accumulation for sorbed ■ (Fe_{sorb}) and precipitated ● (Fe_{ppt}) iron.

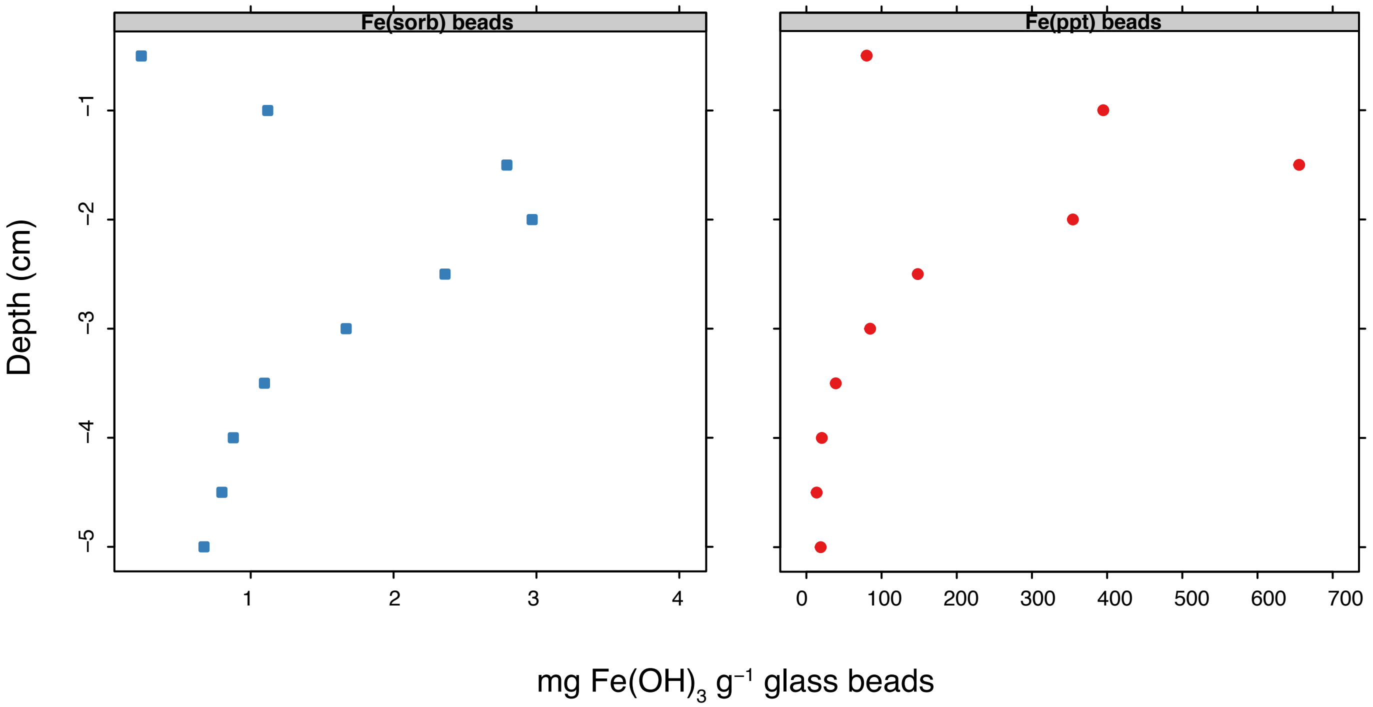

Supplementary Figure 11. Top image shows *Synechococcus* PCC 7002 cells present in aqueous phase. Bottom image shows red autofluorescent cells adhered to glass beads.

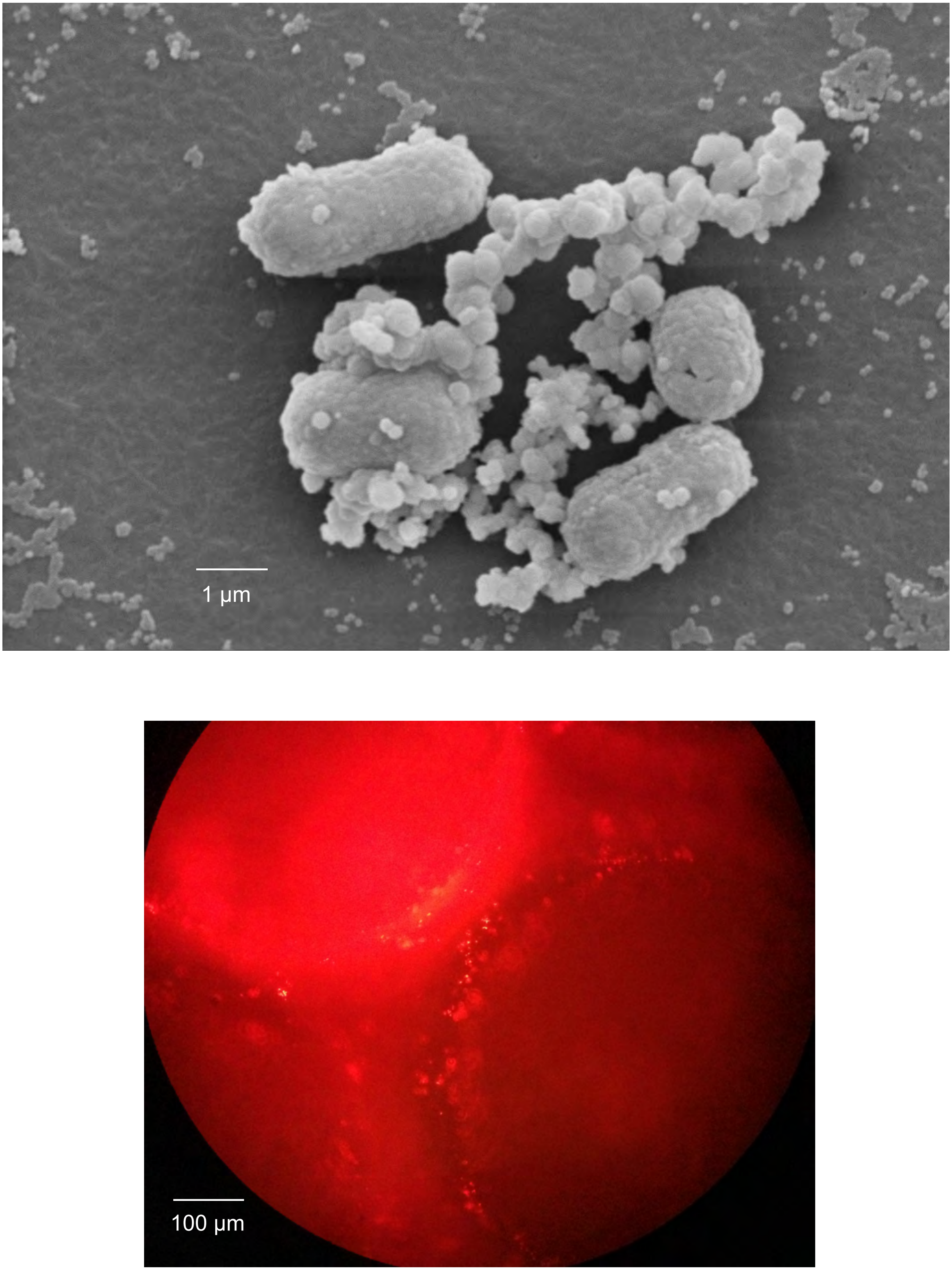

Supplement: Supplementary file 1 — Supplementary Information [file 41598_2018_22694_MOESM1_ESM.pdf]
